# Associations of cardiorespiratory fitness and exercise with brain white matter in healthy adults: A systematic review and meta-analysis

**DOI:** 10.1007/s11682-022-00693-y

**Published:** 2022-06-30

**Authors:** Suzan Maleki, Joshua Hendrikse, Yann Chye, Karen Caeyenberghs, James P. Coxon, Stuart Oldham, Chao Suo, Murat Yücel

**Affiliations:** 1 BrainPark, Turner Institute for Brain and Mental Health, School of Psychological Sciences and Monash Biomedical Imaging Facility, Monash University, 770 Blackburn RD, Clayton, VIC 3168 Australia; 2 Movement and Exercise Neuroscience Laboratory, Turner Institute for Brain and Mental Health, School of Psychological Sciences and Monash Biomedical Imaging Facility, Monash University, Clayton, Australia; 3 Cognitive Neuroscience Unit, School of Psychology, Deakin University, Geelong, Australia; 4 Neural Systems and Behaviour, Turner Institute for Brain and Mental Health, School of Psychological Sciences and Monash Biomedical Imaging Facility, Monash University, Clayton, Australia; 5 Developmental Imaging, Murdoch Children’s Research Institute, Melbourne, Australia

**Keywords:** Exercise, Physical activity (PA), Physical fitness (PF), Cardiorespiratory fitness (CRF), White matter (WM), Magnetic resonance imaging (MRI)

## Abstract

Magnetic resonance imaging (MRI) studies have revealed positive associations between brain structure and physical activity, cardiorespiratory fitness, and exercise (referred to here as PACE). While a considerable body of research has investigated the effects of PACE on grey matter, much less is known about effects on white matter (WM). Hence, we conducted a systematic review of peer-reviewed literature published prior to 5^th^ July 2021 using online databases (PubMed and Scopus) and PRISMA guidelines to synthesise what is currently known about the relationship between PACE and WM in healthy adults. A total of 60 studies met inclusion criteria and were included in the review. Heterogeneity across studies was calculated using Qochran’s q test, and publication bias was assessed for each meta-analysis using Begg and Mazumdar rank correlation test. A meta-regression was also conducted to explore factors contributing to any observed heterogeneity. Overall, we observed evidence of positive associations between PACE and global WM volume (effect size (Hedges’s g) = 0.137, *p* < 0.001), global WM anomalies (effect size = 0.182, *p* < 0.001), and local microstructure integrity (i.e., corpus callosum: effect size = 0.345, *p* < 0.001, and anterior limb of internal capsule: effect size = 0.198, *p* < 0.001). These findings suggest that higher levels of PACE are associated with improved global WM volume and local integrity. We appraise the quality of evidence, and discuss the implications of these findings for the preservation of WM across the lifespan. We conclude by providing recommendations for future research in order to advance our understanding of the specific PACE parameters and neurobiological mechanisms underlying these effects.

**Supplementary Information:**

The online version contains supplementary material available at 10.1007/s11682-022-00693-y.

## Introduction

Engaging in regular physical activity is associated with numerous health benefits, including reduced incidence of certain cancers, cardiovascular disease, and type-2 diabetes (U.S. Department of Health and Human Services, 2019; Australian Department of Health, 2021). Remarkably, the positive effects of exercise also extend to the brain, with large scale epidemiological studies demonstrating that higher levels of physical activity, cardiorespiratory fitness, and exercise (referred to here as ‘PACE’) are associated with a significant reduction in the risk of mild cognitive impairment and dementia in later life (Mandolesi et al., 2018; Stigger et al., 2019). Underlying these effects, a considerable body of research has shown that exercise has profuse, broad effects on neuroplasticity – the brain’s intrinsic ability to modify its structure and function in line with changing internal or environmental factors (Voss et al., 2013). For example, engaging in cardiovascular exercise promotes the release of growth hormones and neurotrophic factors (such as brain-derived neurotrophic factor) that mediate neuroplasticity and are directly implicated in learning and memory (Alkadhi, 2018; Hendrikse et al., 2017).

Here, we use the acronym PACE to encompass any form of physical activity (PA), physical fitness (PF) (i.e., cardiorespiratory fitness; CRF), and exercise intervention. These terms are interrelated and are sometimes used interchangeably, but in fact have distinct definitions. PA can be defined as any bodily movements produced by skeletal muscles and requires energy expenditure, with exercise being a subset of physical activity that has planned, structured, and repetitive movements with a goal of maintaining or improving fitness (Caspersen et al., 1985). While PF is multi-factorial, cardiorespiratory and muscular components are the most commonly assessed, and can be quantified with health or performance measures that index the efficiency of the cardiovascular and respiratory systems. The gold standard method to assess cardiorespiratory fitness (CRF) is to measure the highest rate of oxygen consumption by muscles (known as V ${\mathrm{O}}_{2}$ max) during exercise by maximal exercise test (Campbell et al., 2013; Bouchard et al., 2012).

Higher levels of physical activity, exercise, and cardiorespiratory fitness (i.e. PACE) have beneficial effects on brain volume and integrity (Firth et al., 2018; Sexton et al., 2016). For example, neuroimaging studies have reported positive associations between cardiorespiratory fitness (CRF) and gray matter volume in the hippocampus (Den Ouden et al., 2018), prefrontal cortex, anterior cingulate cortex, and striatum (Firth et al., 2018; Gujral et al., 2017). Similarly, exercise has been associated with improvements in white matter (WM), particularly in older adults (Sexton et al., 2016). WM is composed of myelinated axons, oligodendrocytes, and astrocytes and accounts for approximately half of total brain volume (Sampaio-Baptista & Johansen-Berg, 2017). The primary function of WM is to structurally connect cortical and subcortical regions into ensembles that support cognition. Therefore, optimal coordination, coherence, and conduction velocity of neural activities across different cortical regions are essential for proper cognitive function (Filley & Fields, 2016). WM health can be examined through structural MRI techniques by measuring WM volume (T1-weighted), WM anomalies (T2-weighted), and WM microstructure (e.g., diffusion weighted imaging).

WM anomalies observable as white matter hyperintensities (WMH) in T2-weighted (FLAIR) MRI scans indicate poor WM health. These WMH occur due to water accumulation, reflecting demyelination and axonal loss and are mainly caused by cerebral small vessel disease (Filley & Fields, 2016; Prins & Scheltens, 2015). Aging and poor cardiovascular health (e.g. chronic hypertension and high heart rate) are major risk factors for onset and severity of WMHs (Fuhrmann et al., 2019; Prins & Scheltens, 2015). Mounting evidence demonstrates that WMHs can increase the risk of cognitive impairment (Filley & Fields, 2016; Frey et al., 2019; Fuhrmann et al., 2019; Prins & Scheltens, 2015), dementia (Fuhrmann et al., 2019; Prins & Scheltens, 2015), stroke, and certain forms of mental illness, such as depression (Frey et al., 2019). Further, disruptions in WM integrity (e.g. white matter volume and plasticity) underlie a range of neurodevelopmental, psychiatric, and neurological conditions including autism, schizophrenia, obsessive compulsive disorder, depression, and Alzheimer’s disease (Filley & Fields, 2016). Hence, there is a critical need to investigate methods of maintaining/improving WM integrity throughout the lifespan. Increasing physical activity and/or exercise may provide a novel effective approach, though a comprehensive understanding of the corresponding effect on white matter is first required.

This review aims to provide a systematic review on MRI studies investigating the associations between WM and physical activity, cardiorespiratory fitness and exercise (PACE) in healthy populations.  Again, to maintain a standard terminology throughout this review, we use the term PACE to encompass any form of physical activity (PA), physical fitness (PF) (i.e., cardiorespiratory fitness; CRF), and exercise intervention. A previous review by Sexton et al (2016) highlighted positive associations between higher CRF and WM volume and integrity in frontal and temporal brain regions. However, at the time of publication this review reported cautious support for a link between physical activity and WM outcomes due to the limited evidence base, and only included studies conducted on older adults above 60 years of age. Since then, many new studies featuring young and middle-aged adult samples have been published, warranting an updated review of this literature. Hence, we review the cross-sectional and longitudinal findings to date on each aspect of structural WM health, including WM volume, WM anomalies, and WM microstructure.

## Methods

### Data source and quality check

**Fig. 1:**
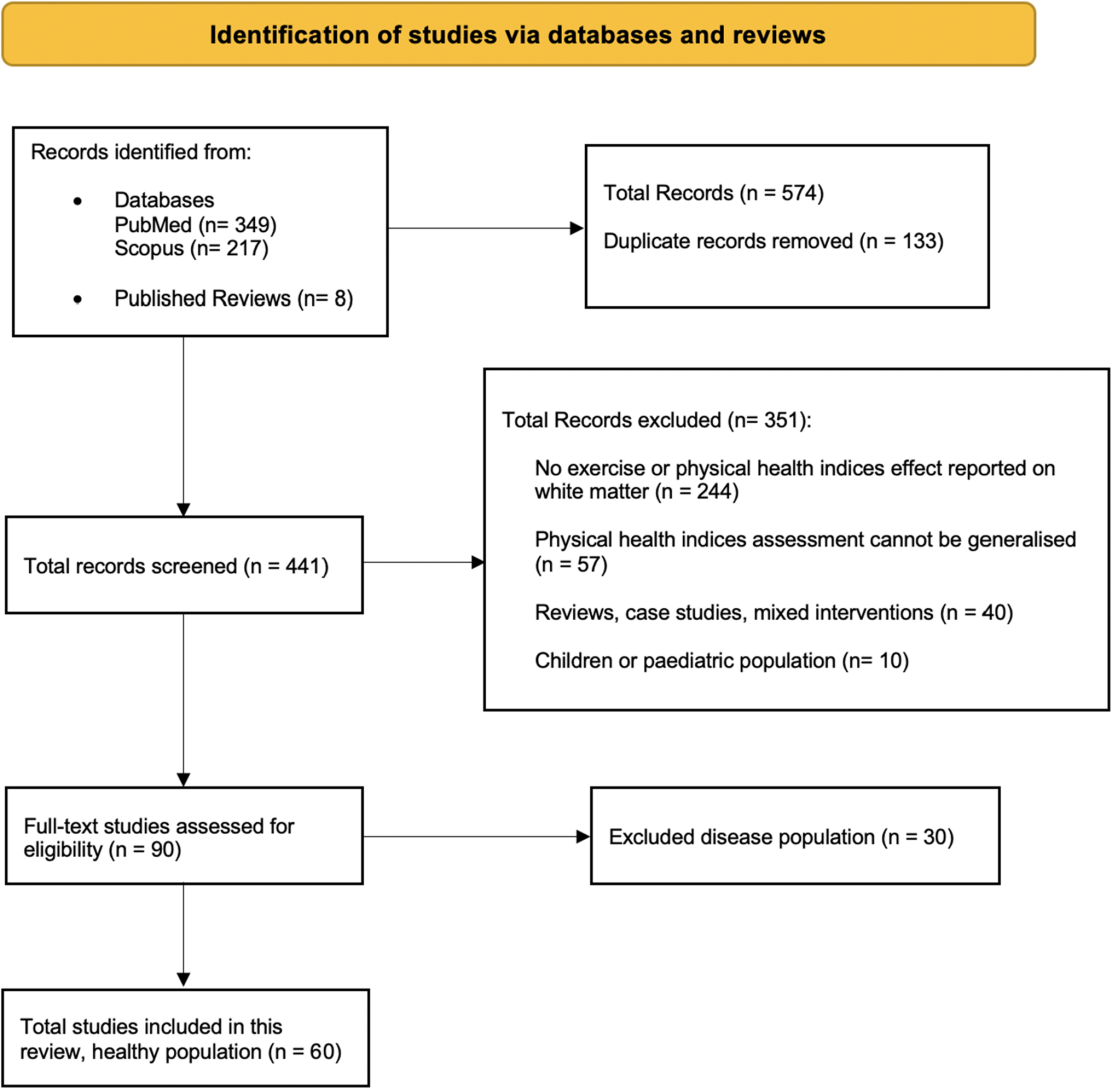
Flow chart depicting the search strategy and number of studies included in the systematic review

The Preferred Reporting Items for Systematic Reviews and Meta-Analyses (PRISMA) framework was used to extract data and report study outcomes (Page et al., 2021). Authors S.M and Y.C conducted a systematic search of the literature via PubMed and Scopus online databases. The search was conducted using the following keywords and operands: “exercise” OR “physical activity” OR “physical fitness” OR “cardiorespiratory fitness” AND “white matter” AND “MRI”. Reference lists of included studies were also screened. Searches were limited to human studies published prior to the 5^th^ July 2021 in the English language. The search strategy is depicted in Fig. 1. The quality of evidence was assessed for each of the included studies by authors S.M and Y.C (and C.S in the case of inter-rater differences) using NIH study quality assessment tools (see supplementary materials). All eligible studies were deemed to have sufficient quality of evidence for inclusion in this review.

### Study selection

Authors S.M. and Y.C. conducted independent title and abstract screening. Inter-rater differences were resolved through consult of author C.S. Studies were required to meet the following criteria for inclusion in this review: (1) published in a peer-reviewed academic journal; (2) utilised either a cross-sectional or longitudinal study design; (3) assessed PACE using objective/quantifiable methodology that could be generalised to wider population (e.g. VO_2max_ for CRF, and actigraphy/accelerometry or self-report measures for PA); (4) included MRI assessment of WM (i.e. volume, anomalies, hyperintensities, and/or microstructural integrity); (5) conducted with healthy participants above 15 years of age. Studies that did not meet these criteria, and/or were conducted with *N* < 10, or utilised a multi-modal intervention without considering a separate exercise group (e.g., exercise combined with cognitive training) were excluded.

### Data extraction

For each study, the following data were extracted: (1) sample demographics (N, age, biological sex), (2) WM assessment, i.e., volume (WMV), hyperintensities (WMH), and/or WM microstructure (fractional anisotropy (FA), mean diffusivity (MD)); (3) study design (i.e., cross-sectional or longitudinal); (4) PACE assessment (e.g., PA/CRF measure, and where applicable exercise intervention parameters including length, frequency and individual session duration). Studies that employed an exercise intervention but only assessed WM at a single timepoint (i.e., pre- or post-intervention were considered cross-sectional). All measures/results are reported as per original study definitions, with the exception of CRF, which for simplicity refers to both VO_2max_ and other related exercise tests of cardiovascular/respiratory function.

### Meta-analysis

Global WM volume and WM anomalies data form cross-sectional studies were analysed using Comprehensive Meta-Analysis (CMA, version 3) (Borenstein et al., 2013). The statistical outcomes included in the meta-analysis were calculated from the original reports. For studies not providing the statistical results of the regions of interest, we contacted the corresponding authors to retrieve these data to maximum our sample size. Given that this meta-analysis was conducted on correlational outcomes, we computed the correlation coefficients and Fisher’s z scale (based on sample size, and p-values of correlational outcomes) for individual studies. These estimates were then used to calculate effect size estimates (Hedges’s g), which provides an unbiased measure of standardised mean differences. We then applied a random-effects model to calculate the total effect size for all the meta-analyses (Borenstein et al., 2010). Similarly, FA measures of WM microstructure from cross-sectional studies were analysed within the most frequently reported regions, namely the corpus callosum and internal capsule (implicated in > 4 studies). Due to insufficient number of studies, a meta-analysis on longitudinal data and cross-sectional global WM microstructure was not performed.

We assessed evidence of heterogeneity across study outcomes using Cochran’s Q method (CMA software) for each performed meta-analysis. Similarly, CMA was used to explore evidence of publication bias using Begg and Mazumdar rank correlations test (Begg & Mazumdar, 1994). We also performed meta-regression analyses to assess the influence of primary subject characteristics (i.e. age and gender) on observed associations between PACE and WM (Hedges’s g). Mean Age and Biological Sex ratios (i.e. % Female) from each individual study sample were extracted and entered as covariates across each meta-analysis model.

## Results

### Overview on selected studies

According to the flow chart (Fig. 1), 60 studies out of 441 articles will be reviewed in this paper. 57 studies were deemed to have good evidence quality, and 3 were fair evidence quality (see supplementary Material). All studies provided a detailed description of their primary study aim(s) and sample demographics, and utilised reliable and valid measures of PACE. Across studies, there was considerable heterogeneity in sample size (longitudinal *N* = 21 – 352; cross-sectional *N* = 15—7148), and exercise intervention parameters. For example, studies utilised different exercise modalities (e.g., walking, cycling, resistance, etc.), intervention durations (i.e., 1 -13 months), frequencies (i.e., 1 – 4 sessions per week), and session durations (20—90 min). MRI scanner field strength also varied across studies (i.e., 1.5 or 3 Tesla). Also, studies varied in their reporting of experimenter blinding and statistical parameters (e.g., p-value specificity), and PACE methodology (e.g., objective vs subjective methods).

### PACE and white matter volume

#### Narrative synthesis

##### Longitudinal studies

Seven studies measured the effects of PACE on WM volume. Five demonstrated significant positive influence of PACE on WM volume (Rehfeld et al., 2018; Tabei et al., 2017; Arnardottir et al., 2016; Best et al., 2015; Stanley J. Colcombe et al., 2006), and two studies did not observe significant effects (Sexton et al., 2020; Smith et al., 2014). Four out of five studies with positive results featured exercise interventions of a minimum six-month duration. However, different types of physical fitness measurements were utilised across studies (e.g., VO_2_max vs self-report measure), and thus it is difficult to conclude whether these effects were directly related to improvements in CRF (please refer to Table 1 for details of longitudinal studies).

**Table 1:** Summary of studies with longitudinal designs

Author	Age (female %)	Sample Size	Intervention Design	Duration (months)	Frequency (times pw)	Session (minutes)	Assessment (MRI and PACE)	Results
Colmenares et al. (2021)	60 – 79 (69.3)	86 51 43	Walking Dance Active control	6	3	60	TP1and TP2: T1/T2 ratio, DTI CRF (treadmill)	**WMH:** ns **Microstructural changes:** Increase total WM (T1/T2 ratio) in genu and splenium of CC (*p* = 0.02), forceps minor (*p* = 0.04), and cingulum (*p* = 0.02) in walking group, relative to controls Increased total WM (T1/T2 ratio) and increased WM in the genu of CC (*p* = 0.05) CRF ~ T1/T2 ratio: ns ↑ FA in fornix and forceps minor in dance group relative to controls **Cognition:** increase in T1/T2 ratio was associated with higher cognitive function in the walking group
Sexton et al. (2020)	60 – 85 (63)	23 23	Cycling Control	3	3	30	TP1and TP2: T1, DTI CRF PA (questionnaire)	**WMV:** ns **Microstructural changes:** No significant group differences in FA, AD, and RD **Cognition:** ns
Lehmann et al. (2020)	18 – 35 (64.5)	15 16	Cycling Balance learning	0.5	7 sessions in total	20	TP1and TP2: T1, DTI CRF (bicycle ergometer) PA (questionnaire)	**Microstructural changes:** FA: ns MD ↓ in exercise group (*p*FWE < 0.05) in the bilateral superior longitudinal fasciculus, bilateral anterior thalamic radiation, bilateral uncinate fasciculus, bilateral inferior fronto-occipital fasciculus, forceps minor, and right corticospinal tract RD: ↓ in exercise group (*p*FWE < 0.05) in the right superior longitudinal fasciculus, right inferior fronto-occipital fasciculus, right anterior thalamic radiation, right uncinate fasciculus, right corticospinal tract, and forceps minor **Cognition:** improved performance of complex motor tasks post intervention
Maltais et al. (2020)	> 70 (60)	106	Assessed exercise patterns associated with regular lifestyle	60	_	_	TP1, TP-mid, TP2: T1, DTI PA (questionnaire)	**Microstructural changes:** FA: ns MD: ↑ in participants with lower PA level in uncinate fasciculus **Cognition:** NA
Clark et al. (2019)	57 – 86 (44)	25	Aerobic	6	3	20—45	TP1and TP2: T1, DTI CRF	**Microstructural changes:** FA: ↓ mean global FA across post intervention MD: ns **Cognition:** NA
Rehfeld et al. (2018)	63 – 80 (51.9)	20 18	Dance training Endurance training	6	2	90	TP1and TP2: T1 CRF (bicycle ergometer)	**WMV:** ↑ in both groups (*p* = 0.001 uncorrected). Specifically, higher WMV in the truncus and splenium of CC in the dance group, and higher WMV in the right occipital and temporal regions **Cognition:** ns
Moon et al. (2018)	> 70 (63.8)	152	Assessed exercise patterns associated with regular lifestyle	36	_	_	TP1and TP2: T1, FLAIR PA (questionnaire)	**WMH:** ns Reduced PA level over three years was associated with WMH progression **Cognition:** NA
Best et al. (2017)	70 – 79 (60)	141	Assessed exercise patterns associated with regular lifestyle	36	_	_	TP1and TP2 (after 10 and 13 years) T1, T2, DTI PA (questionnaire)	**Microstructural changes:** FA: ns AD: Higher PA over 10-year period associated with smaller increase in AD in the inferior longitudinal fasciculus, and parahippocampal and dorsal regions of the cingulum **Cognition:** ↑ PA predicted better global cognitive performance (*p* = 0.01)
Tabei et al. (2017)	> 65 (86.3)	61 51 32	Aerobic Aerobic + Music Control	12	1	60	TP1and TP2: T1 PA (interviewed)	**WMV:** ↑ in both exercise groups compared to controls in the right anterior corona radiata (*p* = 0.001 uncorrected) ↓ WMV in control group post intervention **Cognition:** Improved performance following exercise + music intervention, relative to exercise alone
Burzynska et al. (2017)	60 – 79 (68.7)	49 40 42 43	Dance Walking Walking + Nutrition active control	6	3	60	TP1and TP2: DTI CRF PA (accelerometer)	**Microstructural changes:** Significant changes reported in the Fornix, specifically: FA: ↑ in dance group, while ↓ in walking and control (*p* = 0.001) MD and RD: dance group showed smaller increase relative to other groups (*p* = 0.023 and *p* = 0.007 respectively) **Cognition:** ns
Arnardottir et al. (2016)	~ 79.1 (61)	352	Assessed exercise patterns associated with regular lifestyle	60	_	_	TP1and TP2: T1, T2 PA (accelerometer)	**WMV:** At both time-points, ↑ WMV was associated with ↑ total PA (*p* = 0.03) **Cognition:** NA
Bolandzadeh et al. (2015)	65 – 75 (100)	18 13 15	Light resistance Moderate resistance Balance and toning	12	1 2 2	60	TP1and TP2: T2, PD PA (questionnaire)	**WML:** ↓ WML in moderate resistance group relative to the balance and toning condition (*p* = 0.03) ns difference between light resistance training and balance and toning condition **Cognition:** ns
Best et al. (2015)	65 – 75 (100)	41 37 41	Resistance training twice per week Resistance training once per week Balance and toning	13	2 1 2	60	TP1, TP-mid, TP2: T1 PA (short physical performance battery)	**WMV:** ↓ WMV over 2 years in all groups (*p* = 0.009): 0.8% in twice-weekly resistance training, 1.5% in weekly resistance training, 2% in balance and toning condition **Cognition:** improved memory and executive functions after 2-year follow-up
Smith et al. (2014)	65 – 89	97	Assessed exercise patterns associated with regular lifestyle	18	_	_	TP1and TP2: T1 PA (questionnaire)	**WMV:** ns **Cognition:** NA
Palmer et al. (2013)	24 + -(2), (61)	12 9	Unilateral strength training lower limb Control	1	4	_	TP1and TP2: T1, FLAIR, DTI PA (questionnaire)	**Microstructural changes:** Significant changes in left cortico-spinal tract: MD: ↓ in strength training group (*p* = 0.02) FA: ↑ in strength training group relative to control (*p* = 0.01) **Cognition:** NA
Voss et al. (2013)	55 – 80 (64)	35 35	Walking Stretching	12	3	40	TP1and TP2: T2, DTI CRF	**Microstructural changes:** ↑ CRF was associated with ↑ FA only in walking group in prefrontal (*p* = 0.001), parietal (*p* = 0.005), and temporal regions (*p* = 0.03) AD: ns RD: ns **Cognition:** ↑ CRF ~ improved short-term memory
Colcombe et al. (2006)	60 -79 (55)	29 30	Aerobic Stretching	6	3	60	TP1and TP2: T1 CRF	**WMV:** ↑ WMV in aerobic compared to control group in anterior WM tracts including CC (*p* = 0.05) **Cognition:** NA

*PA* Physical activity; *CRF* Cardiorespiratory fitness; *WMV* White matter volume; *WMH* White matter hyperintensity; *WML* White matter lesion. NA = not applicable (i.e. not measured or only assessed for screening), TP1 = time point at baseline, TP2 = time point at completion of intervention, TP-mid = middle time point (36 months for Maltais et al., 2020, and 12 months for Best et al., 2015). Symbol “ ~ ” means association/ correlation. All p-values are corrected unless otherwise stated. MRI Scanner field strength is 3 T, apart from two studies conducted at 1.5 T (Tabei et al., 2017 and Arnardottir et al., 2016). Regarding biological sex ratios, we averaged across sub-groups in five studies (Colmenares et al., 2021; Tabei et al., 2017; Burzynska et al., 2017; Palmer et al., 2013; Voss et al., 2013; and Colcombe et al., 2006). Non-significant outcomes are denoted as “ns”

##### Cross sectional studies

Seventeen studies have investigated the associations between PACE and WM volume. Nine studies reported significant positive associations (Balbim et al., 2021; Benedict et al., 2013; Demirakca et al., 2014; Erickson et al., 2007; Gow et al., 2012; Gu et al., 2020; Ho et al., 2011; Tian et al., 2015; Zhu et al., 2015), while the remaining eight did not observe any significant outcomes (Bugg & Head, 2011; Colcombe et al., 2003; Gordon et al., 2008; Jochem et al., 2017; Koblinsky et al., 2021; Pentikäinen et al., 2017; Tarumi et al., 2021; Wittfeld et al., 2020). Across studies, PACE was associated with increased WMV within particular regions including posterior cingulate gyrus (Balbim et al., 2021; Demirakca et al., 2014), temporal and parietal (Ho et al., 2011; Tian et al., 2015), corona radiata (Ho et al., 2011), and prefrontal and genu of corpus callosum (CC) (Erickson et al., 2007). Please refer to Table 2 for details of cross-sectional studies.

**Table 2:** Summary of studies with cross-sectional designs

Author	Sample Size	Age	Female Rate %	MRI Protocol	Scanner Field Strength	PA/ CRF Assessment	Results
Palta et al. (2021)	1604	45—64	61	T1 T2 DTI	3 T	PA ($${questionnaire)}^{a}$$	**WMH:** ns **Microstructural changes:** ↑ MVPA at midlife ~ ↑ FA (*p* = 0.021) and ↓ MD (*p* = 0.019) at late life (after 25 years) **Cognition:** NA
Balbim et al. (2021)	34	> 65	56	T1 T2 DTI	3 T	PA (questionnaire)	**WMH:** ns **WMV:** ↑ MVPA ~ ↑ WMV in posterior cingulate (*p* = 0.047) and isthmus cingulate (*p* = 0.044) **Microstructural changes:** PA ~ FA: ns **Cognition:** NA
d’Arbeloff et al. (2021)	801	At 45	48	T1 DTI	3 T	CRF (cycle ergometer)	**Microstructural changes:** FA ~ CRF: ns **Cognition:** NA
Koblinsky et al. (2021)	66	65—85	62	T1	3 T	PA (questionnaire)	**WMV:** ns **Cognition:** ns
Tarumi et al. (2021)	30 aerobic 30 sedentary	45—64	50	T1 DTI	3 T	CRF	**WMV:** ns **Microstructural changes:** Global FA and AD was significantly higher in aerobic group. Regional TBSS results: ↑ FA in the genu of CC, cingulum, fornix, SLF, fronto-occipital fasciculus, uncinate fasciculus, anterior and superior corona radiata, anterior limb of internal capsule, (*p* = 0.042) ↑ CRF ~ ↑ FA and AD in same regions ↑ CRF ~ ↑ RD in brainstem; ↓ RD in the external capsule **Cognition:** NA
Gu et al. (2020)	1443	> 65	63.8	T1 T2	1.5 T	PA (questionnaire)	**WMH:** ns **WMV:** ↑ leisure time PA ~ ↑ total WM and hippocampal volume (*p* = 0.03) **Cognition:** NA
Kim et al. (2020)	35 super agers 55 typical agers	> 60	83	T1 DTI	3 T	PA (FitBit for a week)	**Microstructural changes:** ↑ PA ~ ↑ FA in the body of CC (*p* = 0.04) and ↓ MD (*p* = 0.03) and RD (*p* = 0.01) in left inferior longitudinal fasciculus AD: ns **Cognition:** NA
Johnson et al. (2020)	76	59—77	61.8	T1 T2	3 T	CRF	**WMH:** ↑ CRF ~ ↓ WMH volume in older participants (*p* = 0.04) **Cognition:** NA
Strömmer et al. (2020)	399	18—87	55.4	T1 DTI	3 T	PA (questionnaire)	**Microstructural changes:** ↑ PA ~ ↑ FA preservation in 4 out of 21 ROIs including the genu of CC, uncinate fasciculus, external capsule, anterior limb of internal capsule **Cognition:** ↑ FA in the genu of CC ~ less age-related slowing of cognitive processing
Wittfeld et al. (2020)	2103	21 – 84	52.4	T1	1.5 T	CRF	**WMV:** ns **Cognition:** NA
Raichlen et al. (2019)	7148	40 – 69	57.1	T1 T2	3 T	CRF PA (accelerometer)	**WMH:** ↑ CRF ~ ↓ WMH loads (*p* = 0.002) ↑ MV-PA ~ ↓ WMH loads (*p* = 0.02) MV: moderate-to-vigorous **Cognition:** NA
Opel et al. (2019)	1050	28.8	54.5	T1 DTI	3 T	PF (walking endurance test)	**Microstructural changes:** ↑ PF ~ ↑ FA in widespread clusters including the genu of CC, bilateral SLF, bilateral uncinate fasciculus, bilateral internal and external capsule, CST, and cerebellar peduncles **Cognition:** ↑ PF ~ increased global cognitive function
Williamson et al. (2018)	52	18—40	49	T1 T2 DTI	3 T	PA (VO2)	**WMH:** ↑ CRF associated with ↓ WMH anomalies and ↑ Blood flow & vessel density **Cognition:** NA
Vesperman et al. (2018)	107	40 -65	65.4	T1 T2	3 T	CRF	**WMH:** ↑ CRF ~ ↓ WMH volumes **Cognition:** NA
Gujral et al. (2018)	105	60—80	63	T1 DTI	3 T	PA (questionnaire)	**Microstructural changes:** ↑ FA in CC, anterior thalamic radiation, and superior longitudinal fasciculus predicted higher adherence to PA over 12 months aerobic intervention (*p* = 0.05) **Cognition:** NA
Jochem et al. (2017)	834	25—83	53.5	T1	1.5 T	PA (questionnaire) MRI after 6 years	**WMV:** ns **Cognition:** NA
Pentikäinen et al. (2017)	68	61 -75	42.6	T1	1.5 T	CRF	**WMV:** ns **Cognition:** NA
Bracht et al. (2016)	30	25.5(4.2)	57.5	T1 DTI mcDESPOT	3 T	PA (actigraphy)	**Microstructural changes:** FA: ns; MD: ns; AD: ns; RD: ns ↑ PA ~ ↑ MWF (*p* = 0.007) in the right parahippocampal cingulum (with positive ns trend in fornix) **Cognition:** NA
Smith et al. (2016)	88	65—89	73.8	T1 DTI	3 T	PA (Questionnaire) DTI after 18 months	**Microstructural changes:** ↓ PA ~ ↑ FA in the left fornix and stria terminals MD: ns; AD: ns; RD: ns **Cognition:** NA
Oberlin et al. (2016)	113 (Group 1) 154 (Group 2)	60—81 60—80	_	T2 DTI-G1 (12) DTI-G2 (30)	3 T	CRF	**Microstructural changes:** **Group 1:** ↑ CRF ~ ↑ FA in the CC, fornix, bilateral ACR and anterior internal capsule **Group 2:** ↑ CRF ~ ↑ FA in the CC, left Cingulum, bilateral internal capsule, anterior and superior corona radiata, bilateral superior longitudinal fasciculus ↑ CRF ~ ↓ FA in the bilateral posterior limb of internal capsule (all *p* < 0.05) **Cognition:** ↑ CRF associated with ↑ FA and this in turn was associated with better memory performance
Freudenberger et al. (2016)	877	65 (7.7)	55	T1 T2	1.5 T	CRF	**WML:** ns **Cognition:** ↑ CRF ~ higher global cognitive function
Tian et al. (2015)	146	69.6	58	T1	1.5 T	CRF	**WMV:** ↑ CRF ~ ↑ WMV in temporal and parietal at baseline (*p* = 0.05) **Cognition:** NA
Hayes et al. (2015)	32 young 27 old	18—31 55—82	53.1 55.5	T1 DTI	3 T	CRF	**Microstructural changes:** ↑ FA in young and higher fit old adults compared to lower fit old adults in splenium of CC, posterior corona radiata, sagittal stratum, and right superior parietal regions **Cognition:** NA
Zhu et al. (2015)	565	18 -30	54.3	T1 T2 DTI	_	CRF MRI after 5 years	**WMV:** ↑ CRF ~ ↑ WMV **Microstructural changes:** ↑ CRF ~ ↑ FA **Cognition:** NA
Boots et al. (2015)	315	40 -65	67.9	T1 T2	3 T	CRF (questionnaire) 85 subsets (VO2)	**WMH:** ↑ CRF ~ ↓ WMH volume, (*p* = 0.001) **Cognition:** ↑ CRF ~ better cognitive function
Frederiksen et al. (2015)	282	64—85	58.1	T2 FLAIR	_	PA (questionnaire)	**WMH:** ns **Cognition:** ↑ PA ~ higher executive function, but not memory
Fleischman et al. (2015)	167	60 -96	79	T1 T2	1.5 T	PA (actigraphy)	**WMH:** ns **Cognition:** NA
Burzynska et al. (2014)	88	60—78	66.2	T2 DTI	3 T	PA (accelerometer)	**WML:** ↑ MV-PA ~ ↓ WML (*p* = 0.004) **Microstructural changes:** FA: ns ↑ light PA ~ ↑ FA in temporal lobe (*p* = 0.02) ↑ ${sedentary}^{b}$ ~ ↓ FA in Parahippocampal (*p* = 0.03) **Cognition:** NA
Herting et al. (2014)	34	15 – 18	0	T1 DTI	3 T	CRF PA (actigraphy)	**Microstructural changes:** ↑ CRF ~ ↓ FA in left CST (*p* < 0.05) CRF ~ AD: ns CRF ~ RD: ns **Cognition:** NA
Tian et al. (2014a)	39: Sedentary 148: Life activity 89: Exercise	70—79	58.7	T1 DTI	3 T	PA (self-report time spent walking)	**WMH:** ns **Microstructural changes:** ↑ PA ~ ↓ MD in the medial temporal lobe (*p* = 0.023) and cingulate cortex (*p* = 0.006) PA ~ FA: ns **Cognition:** NA
Tian et al. (2014b)	164	> 80	51.8	T1 FLAIR DTI	3 T	CRF based on 400 m walk as fast as possible	**Microstructural changes:** ↑ CRF ~ ↑ FA in cingulum (*p* = 0.019), and ↓ MD in Hippocampus (*p* = 0.035) and entorhinal cortex (*p* = 0.006) **Cognition:** NA
Wirth et al. (2014)	92	60 -90	63	T1 PET	1.5 T	PA (questionnaire)	**WML:** ↑ PA ~ ↓ WML volumes (*p* = 0.05) **Cognition:** ↑ PA ~ higher global cognitive performance
Demirakca et al. (2014)	95	19 -82	53.6	T1	3 T	PA (questionnaire)	**WMV:** Only in subjects > 40 years old ↑ PA ~ ↑ WMV in the right posterior cingulate gyrus and precuneus (*p* = 0.004), and left posterior cingulate gyrus (*p* = 0.023) **Cognition:** NA
Liu et al. (2012)	9 actives 6 control	60—76	46.6	DTI (21)	3 T	CRF PA (self-report time spent walking)	**Microstructural changes:** ↑ CRF ~ ↑ FA, (voxel-wise correlation, *p* < 0.05) in the internal capsule, genu of CC, and brain stem **Cognition:** NA
Johnson et al. (2012)	26	60—69	53.8	T1 DTI (36)	3 T	CRF	**Microstructural changes:** ↑ CRF ~ ↑ FA and ↓ RD in CC CRF ~ MD: ns CRF ~ AD: ns **Cognition:** NA
Benedict et al. (2013)	331	At 75	49.5	T1	1.5 T	PA (questionnaire)	**WMV:** ↑ PA ~ ↑ total WM volume **Cognition:** ↑ PA was associated with higher memory performance
Gow et al. (2012)	638	At 70	47.3	T1 T2, T2* DTI	1.5 T	PA (questionnaire)	**WMV and WML:** ↑ PA was associated with ↑ WMV and ↓ WML **Microstructural changes:** ↑ FA was associated with ↑ PA (*p* = 0.014) MD: ns **Cognition:** NA
Marks et al. (2011)	8 actives 7 control	60—76	46.6	T1 DTI	3 T	CRF	**Microstructural changes:** ↑ CRF ~ ↑ FA moderately in left middle cingulum (*p* = 0.04) CRF ~ MD: ns **Cognition:** NA
Bugg and Head (2011)	52	55—79	71.1	T1	1.5 T	PA (questionnaire) over the past 10 years	**WMV:** ns **Cognition:** NA
Ho et al. (2011)	226	77.9 (3.6)	57.5	T1	1.5 T	PA (questionnaire)	**WMV:** ↑ PA ~ WMV in corona radiata extending into the parietal-occipital junction (*p* = 0.0002 uncorrected) **Cognition:** NA
Erickson et al. (2007)	54	58—80	100	T1	3 T	CRF	**WMV:** ↑ CRF ~ WMV in prefrontal and genu of CC **Cognition:** ns
Gordon et al. (2008)	20 young 40 old	20 -28 60—81	50 57.5	T1	3 T	CRF	**WMV:** ns **Cognition:** ↑ CRT predicted improved cognitive function
Colcombe et al. (2003)	55	55—79	55.6	T1	1.5 T	CRF	**WMV:** ns **Cognition:** NA

*PA* Physical activity; *CRF* Cardiorespiratory fitness. NA = not applicable (i.e. not assessed), WMV = white matter volume, WMH = white matter hyperintensity, WML = white matter lesion. Symbol “ ~ ” means association/ correlation. All p-values are corrected unless otherwise stated. Non-significant outcomes are denoted by “ns”. Footnotes: a) PA measured at baseline and after 25 years, but MRI only assessed at 25-year timepoint. b) individuals were divided into 3 categories of sedentary, light, and moderate-vigorous based on their PA

#### Meta-analysis

**Fig. 2:**
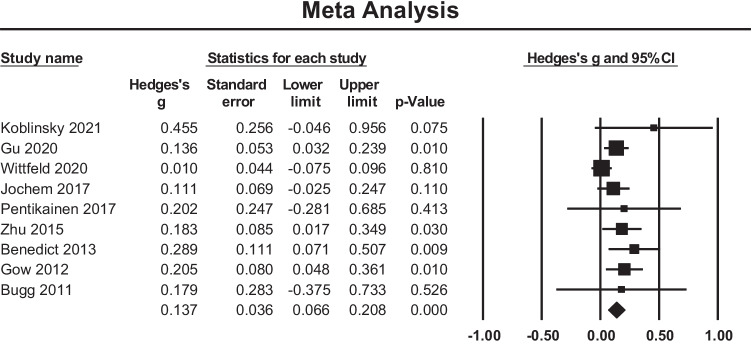
Effect sizes for global white matter volume within cross-sectional studies. Higher PACE is correlated with higher WM volumes

**Fig. 3:**
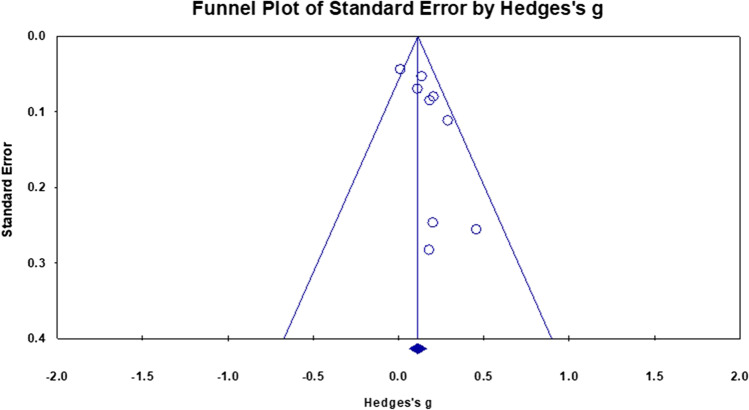
Funnel plot of standard errors plotted against effect sizes (Hedges’s g) for studies in Fig. 2 to visualise publication bias

A meta-analysis of nine cross sectional studies examining the association between PACE and global WMV changes showed an overall small mean effect size of 0.137 (95% confidence interval (CI) = 0.066 to 0.208, *p* < 0.001) (Fig. 2). Studies were not significantly heterogeneous (Q = 12.199, p = 0.143, ${\mathrm{I}}^{2}$= 34.419). The possibility of publication bias was explored by inspecting a funnel plot (Fig. 3) and quantified by calculating Begg and Mazumdar rank correlation test. Qualitatively, there was some evidence of skew in the distribution, though this was not statistically significant (Tau = 0.25, two-tailed p = 0.348). There was also no evidence that the effect size (Hedges’s g) was influenced by sample characteristics (i.e.Age and Biological Sex) (Q = 2.78, df = 2, p = 0.24).

### PACE and white matter anomalies

#### Narrative synthesis

##### Longitudinal studies

Three studies examined the effect of PACE on WMH, with two non-significant results (Colmenares et al., 2021; Moon et al., 2018). One study reported significantly decreased WMH following moderate intensity resistance training (Bolandzadeh et al., 2015). Moon et al. (2018) did not observe significant positive associations, though higher WMH were observed in individuals who engaged in less PACE over a three year follow-up period (Moon et al., 2018).

##### Cross sectional studies

Fifteen studies examined associations between PACE and WMH (Table 2). Nine studies showed significantly reduced WMH in individuals with higher PACE (Boots et al., 2015; Burzynska et al., 2014; Freudenberger et al., 2016; Gow et al., 2012; Johnson et al., 2020; Raichlen et al., 2019; Vesperman et al., 2018; Williamson et al., 2018; Wirth et al., 2014). However, six studies did not observe any significant relation between PACE and WMH (Palta et al., 2021; Balbim et al., 2021; Gu et al., 2020; Frederiksen et al., 2015; Fleischman et al., 2015; Tian et al., 2014a). In summary, while longitudinal evidence is preliminary, the majority of existing cross-sectional studies suggest that greater PACE is associated with a reduced occurrence of WM anomalies.

#### Meta-analysis

**Fig. 4:**
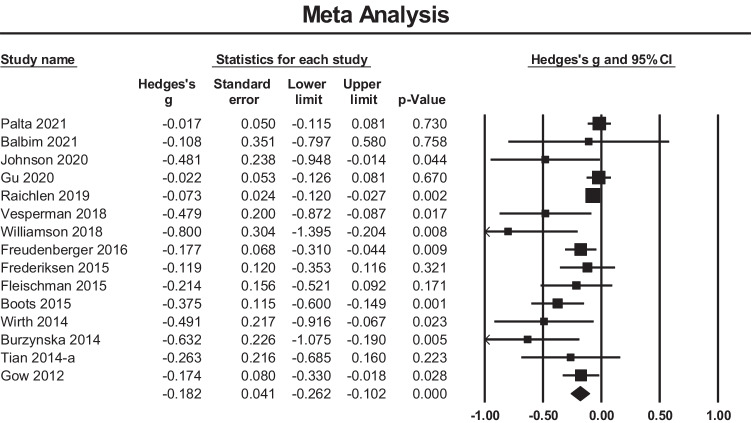
Effect sizes for WM anomalies within cross sectional studies. Higher PACE is correlated with a reduced occurrence of WM anomalies (hyperintensities)

**Fig. 5:**
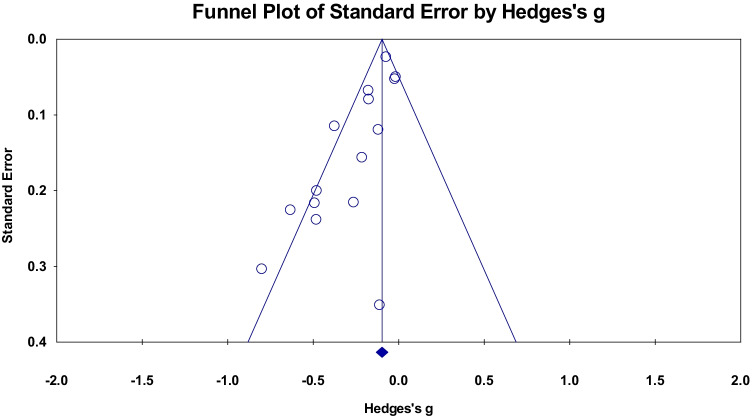
Funnel plot of standard errors plotted against effect sizes (Hedges’s g) for studies in Fig. 4 to visualise publication bias

A meta-analysis of fifteen cross sectional studies examining the relationship between PACE and global WMH volume showed an overall small mean effect size of -0.182 (95% confidence interval (CI) = -0.262 to -0.102, *p* < 0.001) (Fig. 4). There was significant heterogeneity among the included studies (Q = 35.44, p = 0.001, ${\mathrm{I}}^{2}$= 60.50). The funnel plot (Fig. 5) was not symmetric and the Begg and Mazumdar rank correlation was non-significant (Tau = -0.36, two-tailed p = 0.06). The covariates of Age and Biological Sex were entered into the regression model to assess their influence on the observed heterogeneity, however, this was not significant (Q = 0.52, df = 2, p = 0.769).

### PACE and white matter microstructural changes

#### Narrative synthesis

##### Longitudinal studies

Nine studies investigated the effect of PACE on WM microstructure, with the majority utilising DTI outcome measures (Table 1). Basic standard metrics of diffusion analysis are fractional anisotropy (FA), mean diffusivity (MD), axial diffusivity (AD), and radial diffusivity (RD). The two most frequently reported metrics are FA and MD which generally reflect WM integrity and average diffusivity respectively (Curran et al., 2016). Also, increased AD (diffusivity along principal axis) has been linked to axonal damage and increased RD (average of diffusivity along perpendicular axes) has been associated with demyelination (Curran et al., 2016; Mayo et al., 2019).

Three studies found significant FA increase following exercise intervention, while four studies did not find significant effects (Best et al., 2017; Lehmann et al., 2020; Maltais et al., 2020; Sexton et al., 2020). Of those studies reporting positive effects, FA increases were observed across a number of brain regions including prefrontal, parietal, and temporal cortices (Voss et al., 2013), as well as specific WM tracts including the fornix (Burzynska et al., 2017), and left corticospinal tract (CST) (Palmer et al., 2013). Interestingly, one study reported a decrease in whole brain mean FA following 6 months aerobic exercise intervention, but these results may have been influenced by demographic differences which were not controlled for between groups (Clark et al., 2019).

The effect of PACE on MD has been analysed in five studies. Four studies reported significant associations between PACE and MD (Burzynska et al., 2017; Lehmann et al., 2020; Maltais et al., 2020; Palmer et al., 2013), while one study reported no significant effects (Clark et al., 2019). Three studies reported changes in MD following exercise interventions across a number of tracts including superior longitudinal fasciculus, anterior thalamic radiation, uncinate fasciculus, inferior fronto-occipital fasciculus, forceps minor, and the corticospinal tract (Burzynska et al., 2017; Lehmann et al., 2020; Palmer et al., 2013). However, the direction of MD change was inconsistent across studies, with studies reporting both increased (Burzynska et al., 2017) and decreased MD (Lehmann et al., 2020; Palmer et al., 2013) following exercise. One observational study reported greater decrease in MD over a five year period in individuals engaging in lower PACE (Maltais et al., 2020).

Four studies investigated the effect of PACE on RD. Two studies reported non-significant results (Sexton et al., 2020; Voss et al., 2013), and two studies reported significant outcomes (Burzynska et al., 2017; Lehmann et al., 2020), though the direction of these effects differed between studies. Specifically, one study reported decreased RD in right frontotemporal fiber tracts following exercise (Lehmann et al., 2020), while another study found that a dance-based intervention ameliorated the increase in RD observed over a 6-month period in older adults (Burzynska et al., 2017).

Three studies measured the effects of PACE on AD. One study found that higher PACE offset an increase in AD across inferior longitudinal fasciculus, parahippocampal and dorsal regions of the cingulum in individuals over a 10-year period (Best et al., 2017). Two other studies reported no change in AD following an exercise intervention (Sexton et al., 2020; Voss et al., 2013).

One study utilised the T1/T2 ratio (a measure of WM integrity derived by dividing the T1-weighted image by the T2-weighted image) to investigate the effect of a 6-month aerobic exercise on WM integrity. Significant differences in total WM were observed in a walking group relative to controls, with increases observed in the genu and splenium of CC, cingulum, and forceps minor in the walking group. Similarly, significant differences in total WM were also observed in a dance group relative to controls, with increases observed in the genu of CC following a dance intervention (Colmenares et al., 2021).

##### Cross sectional studies

The association between PACE and WM microstructure has been explored in 21 studies to date. Of these, fifteen studies reported significant associations between PACE and FA (Table 2), while four studies did not observe significant associations (Balbim et al., 2021; d’Arbeloff et al., 2021; Bracht et al., 2016; Tian et al., 2014a). Of the studies reporting significant associations, greater engagement in PACE was correlated with higher FA across numerous regions, particularly the CC (Hayes et al., 2015; Johnson et al., 2012; Kim et al., 2020; Liu, 2012; Oberlin et al., 2016; Opel et al., 2019; Strömmer et al., 2020; Tarumi et al., 2021), anterior limb of internal capsule (Liu et al., 2012; Oberlin et al., 2016; Opel et al., 2019; Strömmer et al., 2020; Tarumi et al., 2021), cingulum (Tarumi et al., 2021; Oberlin et al., 2016; Tian et al., 2014b; Marks et al., 2011), uncinate fasciculus (Opel et al., 2019; Strömmer et al., 2020; Tarumi et al., 2021), and superior longitudinal fasciculus (Oberlin et al., 2016; Opel et al., 2019; Tarumi et al., 2021). However, three studies have reported that higher PACE was associated with reduced FA across the bilateral posterior limb of internal capsule (Oberlin et al., 2016), left fornix and stria terminals (Smith et al., 2016), and left CST (Herting et al., 2014).

Correlation between PACE and MD have been reported in nine studies. Overall, four studies reported significant outcomes, while five studies did not observe any significant results (Bracht et al., 2016; Gow et al., 2012; Johnson et al., 2012; Marks et al., 2011; Smith et al., 2016). Of the studies reporting positive outcomes, one study reported significantly lower MD in middle-aged subjects engaging in moderate to vigorous PA, compared to controls (Palta et al., 2021). Two studies observed significant negative correlations between PACE and MD with associations observed across the hippocampus and entorhinal cortex (Tian et al., 2014b), and left inferior longitudinal fasciculus (Kim et al., 2020). One study compared MD between sedentary individuals and those engaging in regular exercise. Significant differences in MD were observed between groups, with lower MD observed in the medial temporal lobe and cingulate cortex in individuals engaging in regular exercise (Tian et al., 2014a).

The association between PACE and AD was examined in six studies. One study reported higher AD in a sample of physically active subjects, relative to controls, across several regions including CC, SLF, uncinate fasciculus, and fornix (Tarumi et al., 2021). However, five studies reported non-significant results (Bracht et al., 2016; Herting et al., 2014; Johnson et al., 2012; Kim et al., 2020; Smith et al., 2016).

Six studies examined the relationship between PACE and RD. Two studies reported significant negative correlations between PACE and RD in corpus callosum (Johnson et al., 2012), and left inferior longitudinal fasciculus (Kim et al., 2020). Of these, however, one study reported inconsistent effects, whereby increased RD was observed in the brainstem and decreased RD in external capsule in physically active subjects (Tarumi et al., 2021). Three studies reported non-significant findings (Bracht et al., 2016; Herting et al., 2014; Smith et al., 2016).

One study employed a multicomponent-driven equilibrium single pulse observation of T1 and T2 (mcDESPOT) sequence to investigate the relationship between PACE and WM integrity. They observed a positive association between PACE and myelin water fraction in right parahippocampal cingulum and a positive trend in the fornix (Bracht et al., 2016).

#### Meta-analysis

**Fig. 6:**
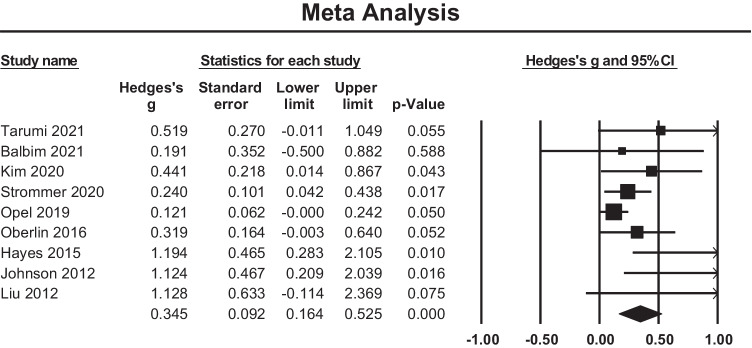
Effect sizes for local WM microstructure reporting FA metric changes in corpus callosum. Higher FA values were positively associated with PACE level

**Fig. 7:**
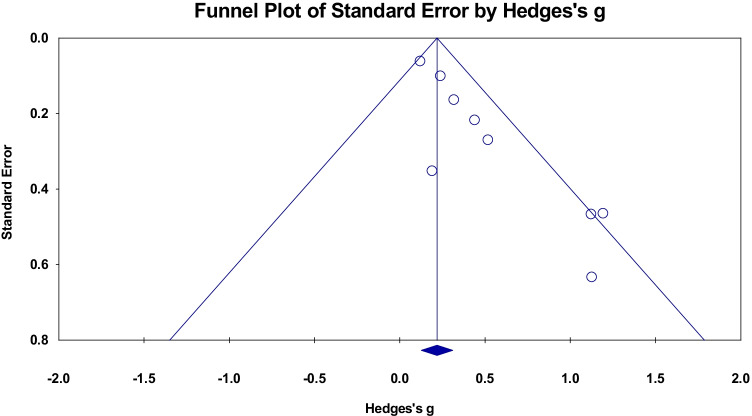
Funnel plot of standard errors plotted against effect sizes (Hedges’s g) for studies in Fig. 6 to visualise publication bias

A meta-analysis of nine cross sectional studies reporting region-of-interest analysis of FA in corpus callosum was conducted. We observed a small positive effect size of 0.345 in the corpus callosum (95% confidence interval (CI) = 0.164 to 0.525, *p* < 0.001, Fig. 6). Studies were not significantly heterogeneous (Q = 15.399, p = 0.052, ${I}^{2}$= 48.05). The funnel plot was partially asymmetric (Fig. 7) and the Begg and Mazumdar rank correlation was significant (Tau = 0.583, two-tailed p = 0.028). The meta-regression showed no significant relationship between Hedges’s g and both covariates (Q = 4.18, df = 2, p = 0.124).

**Fig. 8:**
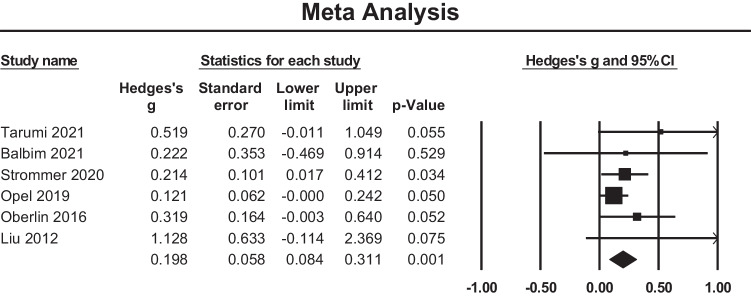
Effect sizes for local WM microstructural findings in anterior limb of internal capsule. Higher FA values were positively associated with PACE level

**Fig. 9:**
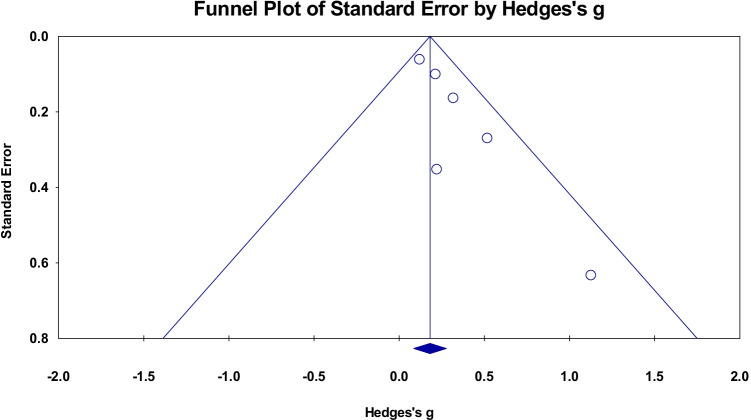
Funnel plot of standard errors plotted against effect sizes (Hedges’s g) for studies in Fig. 8 to visualise publication bias

A meta-analysis of six cross sectional studies reporting FA changes at anterior limb of internal capsule was performed and a small positive effect size of 0.198 was observed (95% confidence interval (CI) = 0.084 to 0.311, *p* < 0.001) (Fig. 8). Studies were not significantly heterogeneous (Q = 5.562, p = 0.351, ${\mathrm{I}}^{2}$= 10.102). The funnel plot (Fig. 9) is symmetric and there was no evidence of significant bias (Tau = 0.53, two-tailed p = 0.132).

## Discussion

We conducted a systematic review of the literature investigating interactions between PACE and WM. We found that majority of cross-sectional and longitudinal studies reported that greater engagement in PACE was associated with greater WM volume and integrity. Similarly, across studies higher PACE was also associated with reduced WM anomalies. This pattern of results was also supported by meta-analysis of the data, which indicated a significant positive effect of PACE on WM volume and integrity, although the size of this effect was small. However, we note that within the sampled literature, several studies reported null results, suggesting that the effects of PACE on WM are likely variable, and quite plausibly influenced by certain methodological considerations and/or PACE parameters. Overall, despite considerable heterogeneity in study methodology and outcomes, we provide evidence of positive correlation between greater engagement in PACE and several aspects of WM. The following sections will provide a detailed discussion of our findings in relation to past evaluations of this evidence, and outline possible methodological variables that may moderate the effects of PACE on WM.

### Evidence of regionally specific effects of PACE on WM

**Fig. 10:**
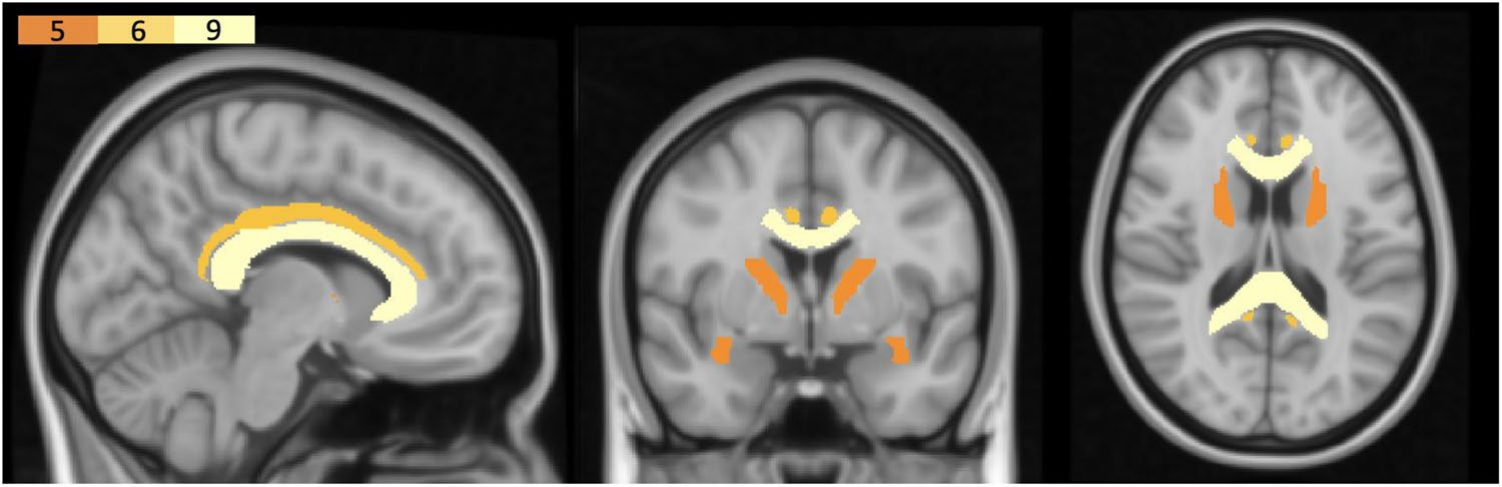
Regions commonly reported in the outcomes of longitudinal and cross-sectional studies. The figure is only for visualisation purposes. The colour bar-plot on top left indicates the number of studies that reported associations between PACE and the WM region

Across the sampled literature, there was some indication of regionally specific interactions between PACE and WM. Specifically, associations between PACE and WM integrity were observed primarily in the corpus callosum (Colmenares et al., 2021; Hayes et al., 2015; Johnson et al., 2012; Kim et al., 2020; Liu et al., 2012; Oberlin et al., 2016; Opel et al., 2019; Strömmer et al., 2020; Tarumi et al., 2021), uncinate fasciculus (Lehmann et al., 2020; Maltais et al., 2020; Opel et al., 2019; Strömmer et al., 2020; Tarumi et al., 2021), internal capsule (Liu et al., 2012; Oberlin et al., 2016; Opel et al., 2019; Strömmer et al., 2020; Tarumi et al., 2021), cingulum (Balbim et al., 2021; Tarumi et al., 2021; Oberlin et al., 2016; Tian et al. 2014a, 2014b; Marks et al., 2011), and fornix (Burzynska et al., 2017; Oberlin et al., 2016; Smith et al., 2016; Tarumi et al., 2021) (see Fig. 10). These findings are largely consistent with those of a previous review by Sexton et al. (2016), which reported some evidence of an association between PACE and WM volume and microstructure (particularly in frontal regions) in older adults. We extend on these findings by demonstrating that these effects are observed across all age cohorts, suggesting that the beneficial effects of PACE are observable across the lifespan.

We also note certain differences between our findings and those of Sexton et al. (2016). Our analysis indicates associations between PACE and WM in temporal regions. Comparatively, Sexton et al. provided some evidence of localised effects within frontal cortex. The cause of these topologically distinct findings is unclear, though it is possible that this may be partially attributable to differences in the age range of sampled literature. The previous review by Sexton et al. (2016) restricted their analysis to studies with a mean sample age > 60 years. Comparatively, we included all studies conducted with healthy adults (i.e. > 18 years of age). While our meta-regression analyses did not show clear evidence of associations between age and primary outcome measures, it remains possible that differences in our sampling approach and inclusion criteria have contributed to these inconsistencies. Frontal and temporal regions are particularly susceptible to age-related structural decline (Bennett & Madden, 2014; Sullivan & Pfefferbaum, 2006), and may therefore be differentially impacted by PACE at different life stages. We aimed to provide a comprehensive assessment of the associations between PACE and WM across the healthy adult lifespan, but future studies are required to elucidate the interactions between PACE, age, and white matter.

### The relationship between exercise ‘dose’ and WM

The optimal exercise parameters for improving WM structure and integrity are yet to be elucidated. It is quite likely that associations between PACE and WM may vary according to the particular exercise parameters under investigation (i.e., modality, frequency, intensity, and duration). While the majority of the literature has investigated the effects of moderate intensity cardiovascular exercise (e.g., moderate intensity walking/cycling), it remains unclear whether these parameters are most effective for improving WM in the healthy population. Similarly, the ideal exercise frequency and duration (e.g., that balance potency and tolerability) are also yet to be established, and thus it is difficult to estimate an ideal ‘dose’ of exercise in this context. Differences in PACE measurement has also likely introduced the consistency in study outcomes. For instance, studies that measured PACE level based on subjective methods (e.g. self-report questionnaires) may have introduced systematic bias into their outcomes compared to studies that employed objective methods (e.g. VO_2_max test). However, given that the links between PACE and WM have been observed across a range of exercise protocols, it is possible that these associations may not relate to the specific nature of the activity per se, but rather depend on the individual’s physiological response to the regimen. In this sense, in developing effective exercise programs to improve WM it may be important to focus on parameters that elicit a certain physiological response (e.g., achieving a certain cardiorespiratory response), to modulate the relevant mechanisms (e.g., brain derived neurotrophic factor (BDNF)) that may mediate these effects. Interestingly, there is some evidence of a positive association between the heart rate response to exercise and BDNF circulation (Marquez et al., 2015). While speculative, focussing on the physiological response to exercise in this manner may offer a means of individualising exercise prescription to maximise associated benefits. Such a framework may also be beneficial in identifying factors which have contributed to the observed variability in study outcomes to date. Future studies are encouraged to report physiological outcomes in response to exercise (e.g., achieved heart rate, VO_2max_, perceived level of exertion) to assess the validity/utility of this perspective.

### Mechanisms mediating the effects of PACE on WM

The underlying mechanisms mediating the effects of PACE on WM remain unclear (Sexton et al., 2016). One plausible hypothesis implicates the known effects of PACE on several cellular and molecular mechanisms mediating aspects of neuroplasticity. For example, both animal and human studies have shown that exercise influences the expression and circulation of key neurotrophins and growth factors (i.e. BDNF, vascular endothelial growth factor (VEGF), and insulin like growth factor (IGF-1)), which modulate a range of microscale structural and synaptic plasticity processes (e.g. synaptogenesis and angiogenesis) (Cotman et al., 2007; Maass et al., 2016). To date, few studies have evaluated the relationship between PACE, WM, and these mechanisms. However, there is preliminary evidence to suggest interactions between expression of these factors and WM (Weinstock-Guttman et al., 2007). Despite the limitations of available measurement techniques in vivo in humans (i.e., reliance on indirect peripheral estimates), future studies are encouraged to investigate the possible mediating role of these factors in the relationship between PACE and WM.

The effects of PACE on WM may also occur via activity-dependent myelination. There is evidence that action potentials trigger the sequence of events underlying myelination (Zatorre et al., 2012). Physical activity and exercise inherently rely upon movement and the distributed brain networks that underpin interlimb coordination (Byblow et al., 2007; Caeyenberghs et al., 2011; Coxon et al., 2010; Swinnen, 2002; Swinnen & Wenderoth, 2004). As such, it is plausible that the neural activity supporting interlimb coordination during physical activity/exercise stimulates myelination processes, which may over time manifest as an overall increase in WM volume and integrity across distributed networks. On a functional level, an increase in myelination in this manner may serve to increase conduction speed across networks supporting interlimb coordination to increase the efficiency/accuracy of movement intrinsic to specific forms of physical activity/exercise. This perspective may also help to explain the regionally specific effects of PACE on WM. Namely, the consistent relationships observed between PACE and certain tracts, such as the corpus collosum and anterior internal capsule, may reflect increased communication across brain networks involving these tracts to support motor coordination, or possibly other cognitive demands during exercise/physical activity (e.g., spatial memory, decision making). While speculative, future studies may also consider this potential relationship between the functional demands inherent to exercise, and associated influence on white matter.

## Conclusion

In summary, following our systematic review of the literature, we report evidence of a significant positive association between PACE and WM within the healthy population. Interestingly, there was evidence of a regionally specific relationship between PACE and WM, with medial temporal regions/tracts commonly reported in study outcomes. Future studies are encouraged to consider/report the physiological response to exercise (e.g. heart rate, and BDNF) to help elucidate potential factors contributing to the heterogeneity in study outcomes and plausibly optimise the prescription of exercise. Future work in this field may also consider the relevance of particular neurotrophic growth factors in mediating neuroplasticity and the relationship between PACE and WM. In regard to MRI methodology, the majority of studies have employed diffusion imaging to investigate correlations between PACE and WM microstructure. Moving forward, studies are recommended to employ multi-modal methods to gain a more nuanced understanding of the specific WM components influenced by PACE. For example, future may employ MRI modalities that are sensitive to changes in myelination, such as the T1/T2 ratio, magnetisation transfer ratio, or mcDESPOT (Sampaio-Baptista & Johansen-Berg, 2017). It is hoped that improving our understanding of the influence of PACE on WM may yield novel, effective lifestyle-based interventions to optimise brain health across the lifespan.

## Supplementary Information

Below is the link to the electronic supplementary material.

Supplementary file1 (XLSX 16 KB)

## Data Availability

Not applicable.
